# Association of Anticardiolipin Antibody in Myocardial Infarction: A Systematic Review and Meta-Analysis

**DOI:** 10.7759/cureus.91474

**Published:** 2025-09-02

**Authors:** Yaaseer Ali Malik, Sanaullah Khan, Salman Habib Roghani, Awais Ahmad Nizami, Mamoon Qadir, Abdul Haseeb, Muhammad Rizwan Umer, Amna Akbar, Alif Hasan Pranto, Tazore Hossen Mahin, Imtiaz Ahmed, Shahad Saif Khandker

**Affiliations:** 1 Cardiology, University Hospital Wales, Cardiff and Vale University Health Board, Cardiff, GBR; 2 Cardiology, Pakistan Institute of Medical Sciences (PIMS), Islamabad, PAK; 3 Cardiology, Cardiff and Vale University Health Board, Cardiff, GBR; 4 Cardiology, Shahida Islam Medical College, Lodhran, PAK; 5 Cardiology, Federal Government Polyclinic, Islamabad, PAK; 6 Internal Medicine, Lady Reading Hospital, Peshawar, PAK; 7 Trauma Surgery, Royal Sussex County Hospital, Brighton, GBR; 8 Trauma and Orthopaedics Surgery, Sarosh Hospital Diagnostic Center, Muzaffarabad, PAK; 9 Pharmacy, Bangladesh Rural Advancement Committee (BRAC) University, Dhaka, BGD; 10 Pharmacology, North South University, Dhaka, BGD; 11 Biochemistry and Molecular Biology, Jahangirnagar University, Dhaka, BGD; 12 Biochemistry and Molecular Biology, Gono Bishwabidyalay, Dhaka, BGD

**Keywords:** anti-cardiolipin antibody, autoantibody, cardiovascular, heart, myocardial, thrombosis

## Abstract

Myocardial infarction (MI) is a serious form of cardiovascular disease (CVD) that can be fatal. On the other hand, antiphospholipid antibodies (aPLs) and their subtypes are found in thrombosis, thrombocytopenia, or other CVDs. Anticardiolipin antibody (ACA), a subtype of aPL, is found in MI and other CVDs; however, the level of ACA in MI compared to the control group was not previously determined in a meta-analysis. In this research, we examined the odds ratio (OR) of ACA in MI patients compared to a healthy control group. After reviewing 180 articles, we selected eight studies and evaluated the OR using a forest plot. We also analyzed the asymmetry and possible outliers, heterogeneity, sensitivity, and quality. Our findings revealed an OR of 4.36 (95%CI: 1.64-11.61; P=0.003), indicating that ACA is found at a higher level in MI patients as compared to healthy controls. The studies were of high quality and exhibited moderate heterogeneity. The OR of 2.26 (95%CI: 1.74-2.94; P<0.00001) from the fixed effect model further supported the main outcome's high sensitivity. ACA can be a useful and feasible biomarker to diagnose and predict the chances of MI. Further research is required to determine an accurate cut-off value of ACA through which the possibility of MI can be predicted in patients with thrombosis, thrombocytopenia, or other related CVDs.

## Introduction and background

According to the World Health Organization, cardiovascular diseases (CVDs) are responsible for approximately 32% of global deaths, amounting to nearly 17.9 million fatalities each year [1,2]. CVD can be caused by the narrowing or blockage of coronary arteries due to the buildup of atherosclerotic plaque, which is generally formed inside the artery [3]. This accumulation impedes blood flow, reducing the delivery of oxygen and essential nutrients to the heart muscle (myocardium). The resulting mismatch between the heart’s oxygen demand and supply can lead to myocardial infarction (MI) or acute myocardial infarction (AMI), which can be referred to as a heart attack, which is a serious form of CVD [4]. MI is primarily triggered by atherothrombosis in the artery, which arises when an atherosclerotic plaque ruptures or erodes, leading to the development of a thrombus that may partially or completely obstruct a coronary artery [5]. MI results in permanent damage to cardiac muscle due to insufficient oxygen supply. It can compromise both diastolic and systolic function and raise the risk of arrhythmias. MI may also lead to various life-threatening complications [6]. Timely reperfusion and restoration of coronary blood flow, particularly within six hours of symptom onset, are critical to improving patient outcomes [7].

Studies have identified a relationship between circulating antiphospholipid antibodies (aPL) and conditions like venous and arterial thrombosis and thrombocytopenia [8]. Anticardiolipin antibodies (ACA) are a subtype of aPL, which represent a heterogeneous group of autoantibodies that target anionic phospholipids, phospholipid-protein complexes, and in some cases, proteins alone [9]. MI is frequently caused by thrombosis in the coronary arteries, where a blood clot blocks blood flow, leading to myocardial cell death [10]. ACA is recognized as one of the markers associated with the occurrence of thrombotic events [11]. These occurrences indicate that the ACA level may rise in cases of MI as well.

Previously, no meta-analysis investigated the correlation of ACA level in MI patients as compared to healthy participants, which might help to assess whether the ACA can be a diagnostic and prognostic biomarker for MI patients with or without autoimmune diseases. Therefore, in this study, we tried to evaluate the correlation between the level of ACA among MI patients and healthy participants, obtaining data from previous studies.

## Review

Methodology

Study Guidelines, Search Method, and Inclusion

This research adhered to the Preferred Reporting Items for Systematic Reviews and Meta-Analyses (PRISMA) guidelines as referenced in earlier studies [12,13]. Searches were conducted across three databases: PubMed, ScienceDirect, and Google Scholar, using targeted keywords like “anticardiolipin antibody”, “ACA”, “myocardial infarction”, and “MI” with specific search strategies. In PubMed, an "advanced" search was performed using "title and abstract" fields, while ScienceDirect utilized the "title, abstract, keywords" option in its advanced search. For Google Scholar, the same keywords were applied using the "allintitle" function. Boolean operators were employed as necessary. Following the search, the authors meticulously identified research articles containing data on the ACA level in MI patients, comparing the level with healthy controls. Articles such as comprehensive reviews, systematic reviews, case reports, letters to the editor, correspondence, and editorials were excluded from this study.

Characteristics and Study Quality

This meta-analysis utilized the event (case) number with a higher level of ACA and the total number of participants for both the MI and healthy control groups, respectively. Additionally, to describe all the selected studies, data were extracted regarding study location, study type, participant demographics (i.e., the percentage of male and female participants), types of case and control group, and the methods used for detecting ACA. The quality of the selected studies was assessed by answering quality-check questions from the National Institutes of Health (NIH) and the University of North Carolina (UNC) [14,15]. If a question was clearly answered (Y), the study received one point; if not addressed (N), it received zero points; if partially answered (P), it received 0.5 points; and if not reported (NR), it received no points. Ultimately, the total points from eight different questions were converted into percentages to determine the overall score. Based on this score, the quality of the selected studies was evaluated following previous studies with slight modifications [16,17]. A percentage score above 70% was considered high quality (low bias risk), below 50% was considered low quality (high bias risk), and scores in between were considered moderate quality (moderate bias risk).

Bias, Meta, and Sensitivity Investigation

To examine study bias, researchers assessed the asymmetry and presence of outlier studies among the selected studies using a funnel plot, as outlined in a previous study [18]. Additionally, heterogeneity was evaluated using the I^2^ value, with I^2^>75% indicating significant heterogeneity, in line with prior research [18,19]. Heterogeneity reflects the differences in study outcomes across various studies [19]. A meta-analysis was conducted with the primary data extracted from the included studies, employing a forest plot with a random effect model. The primary goal was to determine the odds ratio (OR) and the 95% confidence interval (95% CI) through the forest plot. To assess the reproducibility and sensitivity of the main forest plot, another forest plot was generated using a fixed effect model [17,18]. All these analyses were performed using RevMan software (version 5.4) (The Cochrane Collaboration, London, UK).

Subgroup Analysis

Based on the immunoglobulin M (IgM) subtype of ACA among both the event number of MI vs healthy control, along with their total participant number, a subgroup analysis was done using the forest plot with a random effect model.

Results

Study Selection and Characteristics Identification

**Figure 1:**
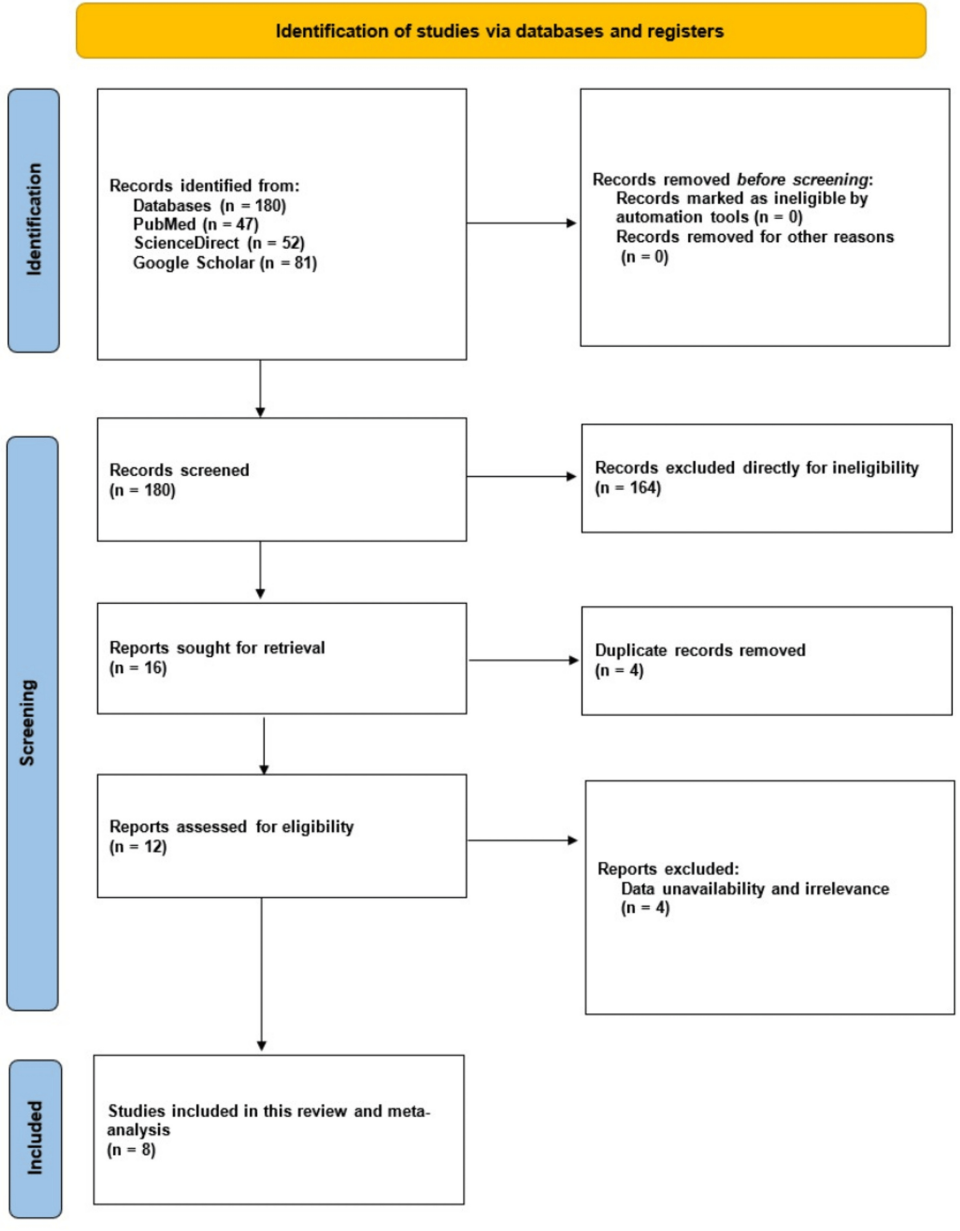
A simplified PRISMA flow diagram of methodology. PRISMA: Preferred Reporting Items for Systematic Reviews and Meta-Analyses

The search strategy yielded a total of 180 articles from three databases: PubMed (n=47), ScienceDirect (n=52), and Google Scholar (n=81). Initially, 164 articles were excluded because they did not qualify as full-length research articles. Out of the 16 remaining articles, four were eliminated due to duplication, and four were excluded for not being relevant to the studies of interest. Ultimately, eight articles were selected for inclusion in this study [20-27]. Figure 1 displays the detailed search and selection process using the PRISMA flow chart (Figure 1).

We conducted a thorough evaluation and extraction of data variables for the study characteristics from the four chosen articles. Table 1 offers a detailed breakdown of the data variables from these selected studies.

**Table 1:** Characteristics of the selected studies ACA: Anticardiolipin antibody, ELISA: enzyme-linked immunosorbent assay, NR: not reported.

Study ID	Location	Study type	Total number of participants		Case Type	Control Type	Age (mean, range)	Method used to detect ACA
			Male, (%)	Female, (%)				
Tahlan et al. 2020 [20]	India	Case-control	82.5	17.5	MI Patient	Healthy control	38.8 (28.6-49)	ELISA
Eber et al. 1990 [21]	Austria	Case-control	100	0	MI Patient	Healthy control	60 (34-87)	ELISA
Brey et al. 2001 [22]	USA	Case-control	NR	NR	MI Patient	Healthy control	56.5 (48–70)	ELISA
Kelishadi et al. 2003 [23]	Iran	Case-control	NR	NR	MI Patient	Healthy control	21.5 (12-55)	ELISA
Zuckerman et al. 1996 [24]	Israel	Case-control	NR	NR	MI Patient	Healthy control	57.5 (50–65)	ELISA
Güler et al. 2000 [25]	Turkey	Case-control	62.5	37.5	MI Patient	Healthy control	58 (NR)	ELISA
Ertaș et al. 2013 [26]	Turkey	Case-control	74	26	MI Patient	Healthy control	59.18 (32–85)	ELISA
Yilmaz et al. 1994 [27]	Turkey	Case-control	85.5	14.5	MI Patient	Healthy control	41.8 (29–50)	ELISA

Quality, Outlier, and Heterogeneity-based Outcome

All the studies selected were of high quality, with scores exceeding 70% except one that scored less than 70% but more than 60% confirming itself as a moderate-quality study. Specifically, three studies scored 88.89%, four studies scored 77.77%, and one scored 66.66% (Table 2). This suggests that there is a low risk of bias in these studies (Table 2).

**Table 2:** Quality assessment of the selected studies. The numbers in columns refer to questions: 1. Was the research question appropriate? 2. Is the target/study population clearly defined? 3. Were any inclusion or exclusion criteria mentioned? 4. Was any time frame mentioned? 5. Are non-responders clearly described? 6. Does the sample represent the target population? 7. Were data collection methods standardized? 8. Was the anti-cardiolipin measuring kit/tool validated? 9. Did the authors use statistical analyses? Y=Yes, N=no, NR=not reported, U=unclear, NA=not applicable. For quality assessment, the “quality assessment tools” of NIH and UNC were used [14,15]. NIH: National Institutes of Health; UNC: University of North Carolina.

Study ID [Reference]	1	2	3	4	5	6	7	8	9	Score %
Tahlan et al. 2020 [20]	Y	Y	Y	Y	Y	Y	Y	Y	N	88.89
Eber et al. 1990 [21]	Y	Y	N	N	Y	Y	Y	Y	Y	77.77
Brey et al. 2001 [22]	Y	Y	N	Y	Y	Y	Y	Y	Y	88.89
Kelishadi et al. 2003 [23]	Y	Y	N	N	Y	Y	Y	Y	Y	77.77
Zuckerman et al. 1996 [24]	Y	Y	N	N	Y	Y	Y	Y	N	66.66
Güler et al. 2000 [25]	Y	Y	Y	N	Y	Y	Y	Y	N	77.77
Ertaș et al. 2013 [26]	Y	Y	Y	N	Y	Y	Y	Y	Y	88.89
Yilmaz et al. 1994 [27]	Y	Y	N	Y	Y	Y	Y	Y	N	77.77

**Figure 2:**
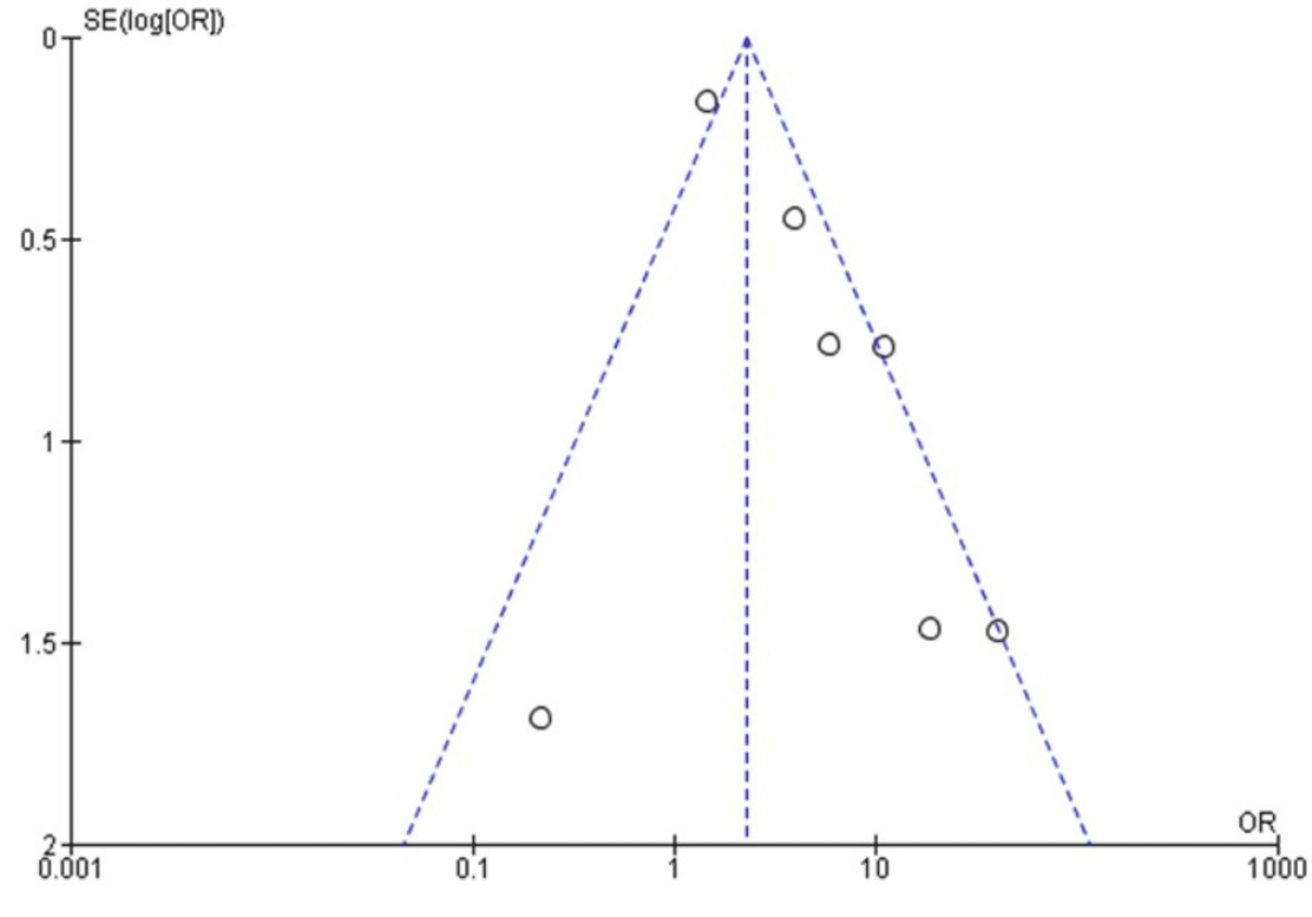
Funnel plot Assessing the possible outlier studies. However, no substantial outlier study was detected.

Additionally, a funnel plot was used to assess study bias, but it did not reveal any significant asymmetry or outliers (Figure 2).

This confirmed that the studies included in our meta-analysis were symmetrical and free from major asymmetry. Interestingly, the heterogeneity among the selected studies was found to be non-significant (<75%), with an I^2^ value of 73%.

Meta-analysis and Sensitivity Reports

**Figure 3:**
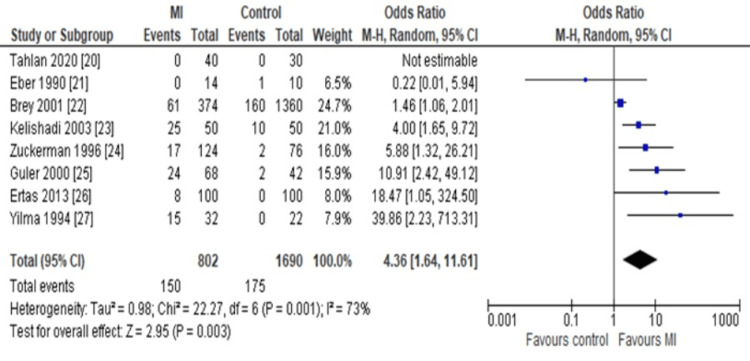
Forest plot Assessing the odds ratio (OR) of anticardiolipin antibody (ACA) immunoglobulin G (IgG) in myocardial infarction (MI) patients compared to healthy controls using the random effect model (REM).

The main forest plot using the random effect model detected the value of the OR as 4.36 (95%CI: 1.64-11.61), which indicated that the ACA was favoring or higher in MI patients as compared to healthy controls (Figure 3).

**Figure 4:**
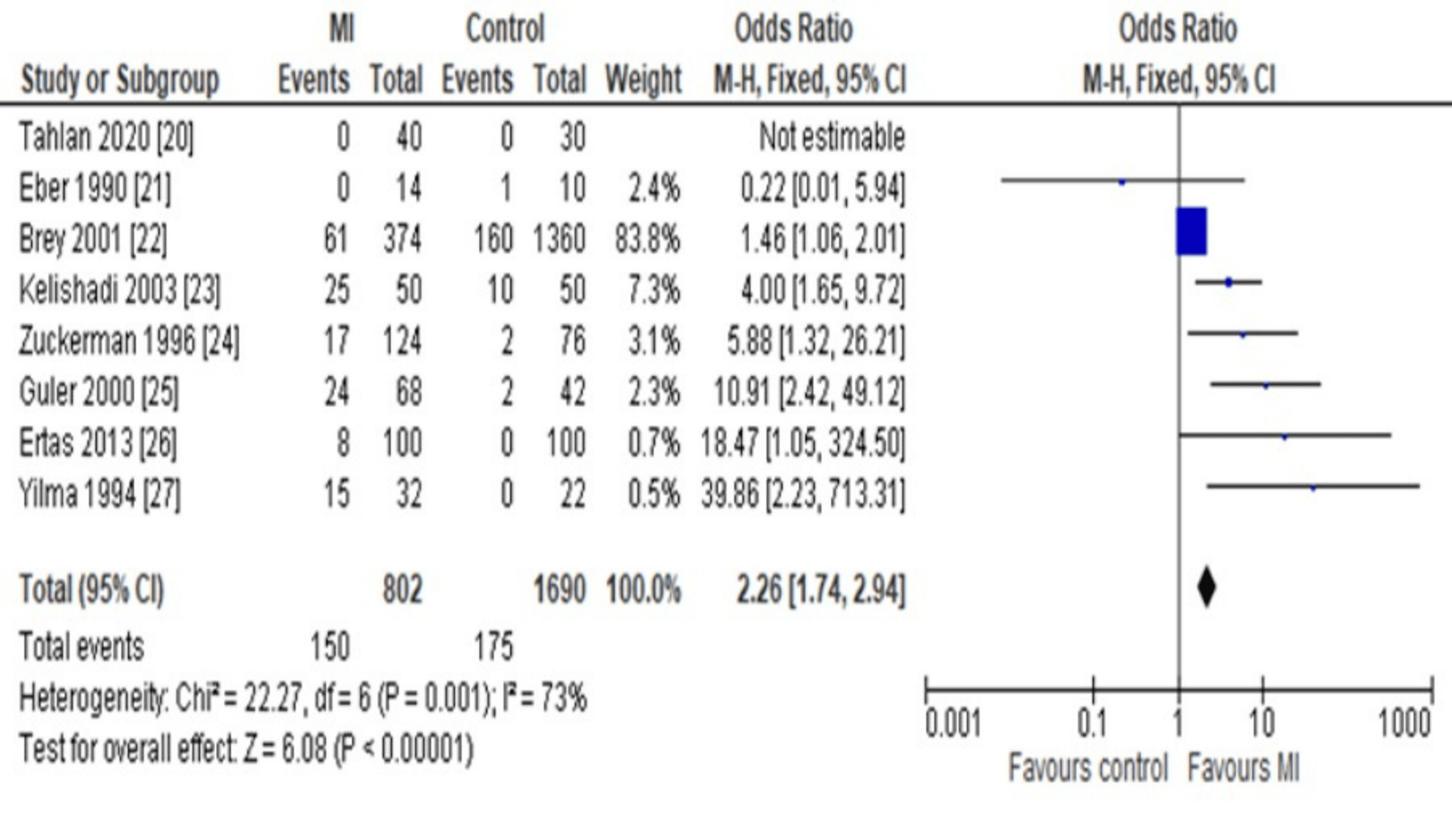
Forest plot Assessing the odds ratio (OR) of anticardiolipin antibody (ACA) immunoglobulin G (IgG) in myocardial infarction (MI) patients compared to healthy controls using the fixed effect model (FEM) for sensitivity analysis. This FEM plot still favors the MI compared to the control as the REM plot.

However, for the re-analysis of the sensitivity and reproducibility of the main meta-outcome, a fixed-effect model was used to regenerate the same forest plot. As a result, the OR was determined as 2.26 (95%CI: 1.74-2.94), which further implied our main outcome to be accurate (Figure 4).

Subgroup-based Findings

**Figure 5:**
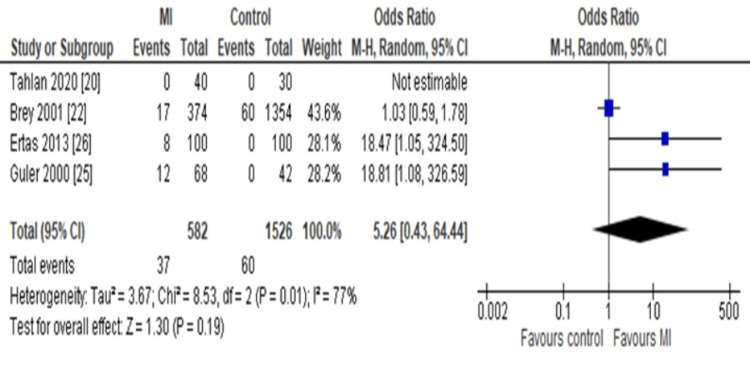
Forest plot Assessing the odds ratio (OR) of anticardiolipin antibody (ACA) immunoglobulin G (IgM) in MI patients compared to healthy controls using random-effect model (REM).

The subgroup analysis was done using a forest plot of random-effects model specifically based on IgM level of the ACA in MI vs healthy control groups. As a result, we determined the OR as 5.26 (95%CI: 0.43-64.44), which also favors the high ACA IgM level in the MI group compared to healthy controls (Figure 5). 

Discussion

Main Outcome-based Discussion

MI continues to be a major cause of death and long-term health complications worldwide. Timely and accurate diagnosis is essential for initiating prompt and aggressive treatment, which can greatly improve survival rates [28]. In this study, which is to the best of our knowledge is the first meta-analysis to thoroughly evaluate ACA levels in MI patients compared to healthy controls, we tried to understand that if ACA can be a prominent biomarker in the diagnosis of MI.

We primarily determined that throughout, all the study participants, patients who suffered MI, exhibited significantly higher levels of aCL antibodies compared to healthy controls (OR: 4.36; 95%CI: 1.64-11.61; P=0.003). According to a study, individuals in the highest quartile of these antibody levels faced a twofold greater risk than the rest of the population, and this risk was independent of conventional coronary risk factors [26].

Interference of ACA with Anticoagulants

Research says that ACA can interfere with natural anticoagulant mechanisms by binding to key regulatory cofactors such as protein C, protein S, annexin V, and β2-glycoprotein I (β2GPI), thereby impairing their function [29,30]. Specifically, IgG of ACA has been shown to stimulate platelet activation and thromboxane production, as well as induce endothelial cell activation, all of which contribute to vascular dysfunction [31]. Besides, ACA predominantly targets phospholipid-binding proteins like β2GPI. Upon binding, they alter β2GPI’s structure, promoting its interaction with cell surfaces such as endothelial cells, platelets, and monocytes [30]. The resulting ACA-β2GPI complexes engage receptors such as annexin A2 and Toll-like receptor 4 (TLR4) on endothelial cells, triggering intracellular signaling that upregulates adhesion molecules and pro-inflammatory cytokines. This promotes leukocyte recruitment and endothelial activation, fostering a prothrombotic environment [32,33].

ACA in Coagulation Activation

In platelets, ACA facilitates activation through receptors like glycoprotein Ibα (GPIbα) and apolipoprotein E receptor 2, enhancing aggregation and the release of prothrombotic agents like thromboxane A2. Similarly, in monocytes, the ACA-receptor interaction via TLR4 upregulates tissue factor (TF) expression and inflammatory cytokines (i.e., IL-1, IL-6, and TNF-α), initiating coagulation and inflammation. ACA can also trigger the classical complement pathway, producing components such as C5a that amplify inflammatory and thrombotic responses. Furthermore, they promote the formation of neutrophil extracellular traps (NETs), which serve as scaffolds for platelet adhesion and carry TF, further supporting clot formation. Finally, ACA can disrupt annexin A5 protective layers on cell membranes, exposing phospholipids and accelerating coagulation [32,33].

ACA in MI

There is a rise in the ACA antibody level after MI, possibly due to an immunological response to tissue necrosis [34,35]. Research-based evidence supports the idea that atherosclerosis is, at least in part, an inflammatory process. It was observed that oxidized low-density lipoprotein (ox-LDL) plays a critical role in this inflammation, and ACA may cross-react with ox-LDL. Furthermore, ACA has been found to enhance the uptake of ox-LDL by macrophages, suggesting a potential role for these antibodies in the development and progression of atherosclerosis [36,37].

Antibodies to cardiolipin are frequently found in young patients following myocardial infarction and may serve as indicators of increased risk for recurrent cardiovascular events [27]. Other research identifies a significant difference in the prevalence of ACA positivity between children of individuals with premature MI and those without such a family history. Additionally, a strong concordance in ACA positivity was observed between affected parents and their children. These findings imply that elevated ACA levels may exist before the clinical onset of MI, supporting the potential role of ACA as an early marker or predictor of myocardial infarction [38-40].

Elevation of ACA IgM

Previously, it was reported that both the IgG and IgM isotypes of ACA are autoantibodies associated with an elevated risk of thrombosis [41]. In subgroup analysis, we found similar results in the case of ACA IgM level elevation among MI patients as compared to healthy controls with an OR of 5.26 (95%CI: 0.43-64.44; P=0.19). A study from Turkey also reported that 8% of patients with AMI tested positive for ACA IgG and 5% for ACA IgM, while none of the control group showed positivity. These findings indicate that both isotypes are elevated in AMI, with IgG appearing more frequently and potentially playing a greater role in the immune response associated with CVDs [26]. Therefore, ACA IgG can be an early diagnostic biomarker. 

Study limitations

We did not explore other websites, search engines, or unpublished materials to gather additional information on ACA data in MI and healthy control patients; instead, we relied solely on data from complete published research articles.

## Conclusions

ACA was determined to be significantly elevated in MI patients as compared to healthy individuals. Therefore, among the various circulating aPLs associated with thrombosis or thrombocytopenia, ACA can be a reliable and feasible biomarker for diagnosing and even predicting MI. Further research is required to determine an accurate cut-off value of ACA through which the possibility of MI can be predicted in patients with thrombosis, thrombocytopenia, or other related CVDs.

## References

[1] Ghodeshwar GK, Dube A, Khobragade D (2023). Impact of lifestyle modifications on cardiovascular health: a narrative review. Cureus.

[2] The International Bank for Reconstruction and Development, The World Bank Global Burden of Disease and Risk Factors: Diseases Control Priorities Project.

[3] McCullough PA (2007). Coronary artery disease. Clin J Am Soc Nephrol.

[4] Shahjehan RD, Sharma S, Bhutta BS (2024). Coronary artery disease. StatPearls.

[5] Ceasovschih A, Mantzouranis E, Dimitriadis K (2024). Coronary artery thromboembolism as a cause of myocardial infarction with non-obstructive coronary arteries (MINOCA). Hellenic J Cardiol.

[6] Reed GW, Rossi JE, Cannon CP (2017). Erratum for acute myocardial infarction, Lancet 2017:389;197-210. Lancet.

[7] Mechanic OJ, Gavin M, Grossman SA (2023). Acute myocardial infarction. StatPearls.

[8] Moulis G, Audemard-Verger A, Arnaud L (2016). Risk of thrombosis in patients with primary immune thrombocytopenia and antiphospholipid antibodies: A systematic review and meta-analysis. Autoimmun Rev.

[9] Tuominen A, Miller YI, Hansen LF, Kesäniemi YA, Witztum JL, Hörkkö S (2006). A natural antibody to oxidized cardiolipin binds to oxidized low-density lipoprotein, apoptotic cells, and atherosclerotic lesions. Arterioscler Thromb Vasc Biol.

[10] Libby P, Ridker PM, Hansson GK (2009). Inflammation in atherosclerosis: from pathophysiology to practice. J Am Coll Cardiol.

[11] Naess IA, Christiansen SC, Cannegieter SC, Rosendaal FR, Hammerstroem J (2006). A prospective study of anticardiolipin antibodies as a risk factor for venous thrombosis in a general population (the HUNT study). J Thromb Haemost.

[12] Islam MA, Khandker SS, Kotyla PJ, Hassan R (2020). Immunomodulatory effects of diet and nutrients in systemic lupus erythematosus (SLE): a systematic review. Front Immunol.

[13] Khandker SS, Nik Hashim NH, Deris ZZ, Shueb RH, Islam MA (2021). Diagnostic accuracy of rapid antigen test kits for detecting SARS-CoV-2: a systematic review and meta-analysis of 17,171 suspected COVID-19 patients. J Clin Med.

[14] (2024). Study quality assessment tools | National Heart, Lung and Blood Institute. https://www.nhlbi.nih.gov/health-topics/study-quality-assessment-tools.

[15] Systematic reviews: step 6: assess quality of included studies | University of North Carolina (UNC). https://guides.lib.unc.edu/systematic-reviews/assess-quality.

[16] Khandker SS, Jahan S, Khan AA (2025). Global epidemiology of HIV among dialysis patients: a systematic review and meta-analysis. Int Urol Nephrol.

[17] Khandker S, Jannat N, Sarkar D (2023). Association between glomerular filtration rate and β-thalassemia major: a systematic review and meta-analysis. Thalassemia Reports.

[18] Mughal HB, Majeed AI, Aftab M (2024). Brain natriuretic peptide in acute heart failure and its association with glomerular filtration rate: a systematic review and meta-analysis. Medicine (Baltimore).

[19] Shabbir NA, Kant SB, Rashid K (2024). Prevalence of HIV/AIDS among pregnant women in North American region: a systematic review and meta-analysis. Medicine (Baltimore).

[20] Tahlan K, Sikka M, Avasthi R, Kotru M, Gogoi P, Gupta R (2020). Antiphospholipid antibodies in young adults with myocardial infarction. Asian J Cardiol Res.

[21] Eber B, Kronberger-Schaffer E, Brussee H (1990). Anticardiolipin antibodies are no marker for survived myocardial infarction. Klin Wochenschr.

[22] Brey RL, Abbott RD, Curb JD, Sharp DS, Ross GW, Stallworth CL, Kittner SJ (2001). Beta(2)-glycoprotein 1-dependent anticardiolipin antibodies and risk of ischemic stroke and myocardial infarction: the Honolulu Heart Program. Stroke.

[23] Kelishadi R, Sabet B, Khosravi A (2003). Anticardiolipin antibody of adolescents and age of myocardial infarction in parents. Med Sci Monit.

[24] Zuckerman E, Toubi E, Shiran A (1996). Anticardiolipin antibodies and acute myocardial infarction in non-systemic lupus erythmatosus patients: a controlled prospective study. Am J Med.

[25] Güler N, Bilge M, Eryonucu B, Erkoç R, Sayarlioğlu M (2000). Relation between left ventricular and/or left atrial thrombus and anticardiolipin antibodies in patients with acute myocardial infarction. Am J Cardiol.

[26] Ertaș F, Can O, Acet H, Ozbakkaloglu M (2013). The clinical significance of anticardiolipin antibody levels in patients with acute myocardial infarction: a regional study. Postepy Kardiol Interwencyjnej.

[27] Yilmaz E, Adalet K, Yilmaz G (1994). Importance of serum anticardiolipin antibody levels in coronary heart disease. Clin Cardiol.

[28] Gravning J, Kjekshus J (2008). The perfect biomarker in acute coronary syndrome: a challenge for diagnosis, prognosis, and treatment. Eur Heart J.

[29] Roubey RA (1998). Mechanisms of autoantibody-mediated thrombosis. Lupus.

[30] Arreola-Diaz R, Majluf-Cruz A, Sanchez-Torres LE, Hernandez-Juarez J (2022). The pathophysiology of the antiphospholipid syndrome: a perspective from the blood coagulation system. Clin Appl Thromb Hemost.

[31] Cuadrado MJ, López-Pedrera C, Khamashta MA (1997). Thrombosis in primary antiphospholipid syndrome: a pivotal role for monocyte tissue factor expression. Arthritis Rheum.

[32] Rand JH (2000). Antiphospholipid antibody-mediated disruption of the annexin-V antithrombotic shield: a thrombogenic mechanism for the antiphospholipid syndrome. J Autoimmun.

[33] Lambert M, Brodovitch A, Mège JL, Bertin D, Bardin N (2024). Biological markers of high risk of thrombotic recurrence in patients with antiphospholipid syndrome: a literature review. Autoimmun Rev.

[34] Vaarala O, Mänttäri M, Manninen V, Tenkanen L, Puurunen M, Aho K, Palosuo T (1995). Anti-cardiolipin antibodies and risk of myocardial infarction in a prospective cohort of middle-aged men. Circulation.

[35] Sletnes KE, Smith P, Abdelnoor M, Arnesen H, Wisløff F (1992). Antiphospholipid antibodies after myocardial infarction and their relation to mortality, reinfarction, and non-haemorrhagic stroke. Lancet.

[36] Vaarala O, Alfthan G, Jauhiainen M, Leirisalo-Repo M, Aho K, Palosuo T (1993). Crossreaction between antibodies to oxidised low-density lipoprotein and to cardiolipin in systemic lupus erythematosus. Lancet.

[37] Shoenfeld Y, Harats D, George J (1998). Atherosclerosis and the antiphospholipid syndrome: a link unravelled?. Lupus.

[38] Wu R, Nityanand S, Berglund L, Lithell H, Holm G, Lefvert AK (1997). Antibodies against cardiolipin and oxidatively modified LDL in 50-year-old men predict myocardial infarction. Arterioscler Thromb Vasc Biol.

[39] Rosove MH, Brewer PM (1992). Antiphospholipid thrombosis: clinical course after the first thrombotic event in 70 patients. Ann Intern Med.

[40] Hamsten A, Norberg R, Björkholm M, de Faire U, Holm G (1986). Antibodies to cardiolipin in young survivors of myocardial infarction: an association with recurrent cardiovascular events. Lancet.

[41] Kelchtermans H, Pelkmans L, de Laat B, Devreese KM (2016). IgG/IgM antiphospholipid antibodies present in the classification criteria for the antiphospholipid syndrome: a critical review of their association with thrombosis. J Thromb Haemost.

